# What Is Really a Nonobstructive Hypertrophic Cardiomyopathy? The Importance of Orthostatic Factor in Exercise Echocardiography

**DOI:** 10.5402/2011/346797

**Published:** 2011-05-23

**Authors:** Carlos Cotrim, Ana Rita Almeida, Luís Lopes, Paula Fazendas, Isabel João, Hélder Pereira

**Affiliations:** Cardiology Department, Hospital Garcia de Orta, Avenida Torrado da Silva, 2805-267 Almada, Portugal

## Abstract

The authors report the case of a 23-year-old girl with nonobstructive hypertrophic cardiomyopathy evaluated by resting echocardiography. The patient complained of syncope after playing basketball. The patient was submitted to treadmill exercise echocardiogram, and she exercised for 9 minutes in standard Bruce protocol. The left ventricular outflow gradient did not occur at peak workload; however she developed intraventricular gradient greater than 100 mmHg after exercise in orthostatic position. There was fall in arterial pressure, and the patient was then put in supine position. The authors suggest the possible role of exercise stress echo in symptomatic patients with no significant gradient at baseline, as well as maintenance in orthostatic position after exercise, as an important stress factor. This can disclose the occurrence of left ventricular outflow tract obstruction that should not be detected in other way and has potential relevance in the patient's symptoms understanding.

## 1. Background

Hypertrophic cardiomyopathy (HCM), a genetic disease cause by mutations in sarcomeric contractile proteins, is characterized by left ventricular (LV) hypertrophy, myocardial fibrosis, and myocyte disarray. As a result, patients may experience functional limitations. Many authors believe that left ventricular outflow tract (LVOT) obstruction is an important pathophysiologic component of the disease and may be one of the mechanisms responsible for symptoms. Its presence at resting conditions is an independent predictor of adverse clinical consequences such as heart failure and sudden death. Besides, the evidence of a significant baseline subaortic gradient may identify patients eligible for more aggressive therapeutic options, namely, surgical myectomy or alcohol septal ablation. 

What if the patient has limiting symptoms and a nonobstructive HCM at resting conditions? The parameters may not reflect daily cardiovascular haemodynamics and the heart's behavior during ordinary activities requiring some physical effort. As the usual evaluation of HCM patients consists of serial resting echocardiography, the pathophysiology during exercise is not taken into account for therapeutic decisions. Because LVOT gradients are dynamic, they may be identified only with exercise, and these findings have already been published by Maron et al. [1] and Shah et al. [2]. Exercise echocardiography should be considered a particularly helpful tool for the assessment of functional capacity and symptom evaluation in this group of patients. This is commonly done at our department [3, 4]. But reviewing the literature, we find that the role of dynamic gradients is still debated [5, 6]. The significance of dynamic outflow gradients is highlighted in this case report, as well as the importance of the *orthostatic position* as an additional stress factor in this condition. 

## 2. Case Report

**Figure 1:**
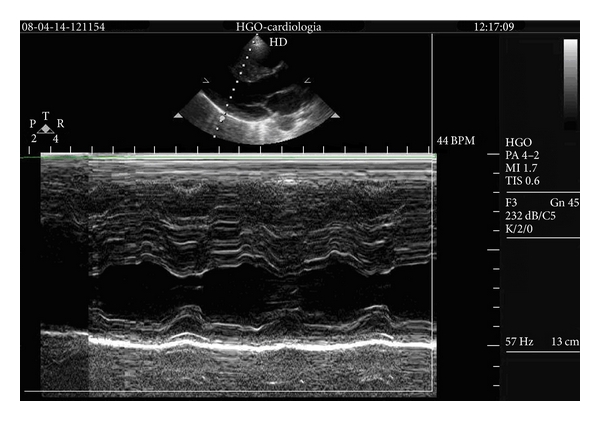
Septal hypertrophy.

We describe the case of a 23-year-old girl with unknown HCM, that has syncope after a basketball game. In the medical evaluation Cardiac auscultation in left lateral decubitus and orthostatic position revealed no systolic murmur, and she did an echocardiogram that reveals HCM. She has septal hypertrophy (Figure 1 and see VIDEO 1 in Supplementary Material available online at doi10.5402/2011/346797), and she does not have systolic anterior movement of mitral valve (SAM) or intraventricular gradient (VIDEO 1 and VIDEO 2). 

**Figure 2:**
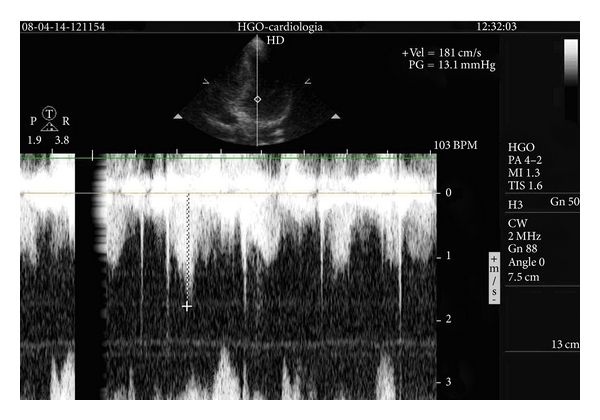
Flow evaluated with continuous wave Doppler at peak exercise demonstrating the absence of LVOT obstruction.

Treadmill exercise echocardiogram was performed, using the standard Bruce protocol, and the patient did not develop LVOT gradient at peak exercise (Figure 2). 

**Figure 3:**
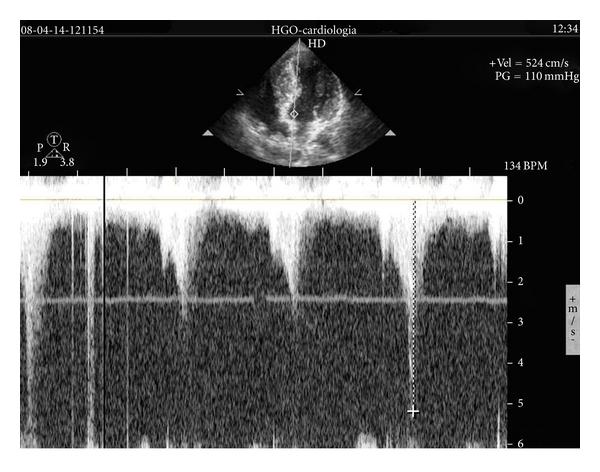
Flow evaluated with continuous wave Doppler after exercise demonstrating the presence of LVOT obstruction.

After finishing the exercise we maintained the patient in orthostatic position and SAM of mitral valve and LVOT gradient greater than 100 mmHg (Figure 3 and VIDEO 3) developed. With evidence of falling systolic arterial pressure from 130 to 110 mmHg in that moment we put the girl in supine position. Previously considered a nonobstructive HCM, after the exercise echocardiography, the patient was considered to have an obstructive pattern with probable influence in clinical symptoms. Therefore, she was medicated with bisoprolol 5 mg a day.

## 3. Discussion and Conclusions

In one study [3] of patients with obstructive HCM at rest, LVOT gradient was increased both in supine and in orthostatic position. This increased gradient in upright position continued to augment at peak exercise, but after exercise, the gradient decreased rapidly as we put patient in supine position. Using the very same methodology Dimitrow et al. [5] in non obstructive HCM patients demonstrates also the importance of orthostatism in disclosing LVOT obstruction in 21% of study group. 

In another study from our group [4] we showed that if after exercise we maintained the patient in orthostatic position the gradient continues to increase during some time and one patient only developed LVOT gradient at this moment. 

The importance of obstruction in HCM prognostic stratification is clearly stated by Maron et al. [6], and the present case reinforces the importance of exercise echocardiography and of orthostatism, before, during, and after exercise as an additional stressor to the occurrence of LVOT obstruction in HCM.

Exercise in upright position may increase the gradient by two mechanisms: decreasing preload by reducing venous return and increasing left ventricular contractility and cardiac output. The sudden end of the activity of muscle activity, of lower limbs, with further decrease of preload, may be the cause of the LVOT gradient that occurs in this phase in some patients.

The other lesson learned from this patient is the usefulness of exercise stress echocardiography in the evaluation of symptomatic, nonobstructive HCM, classified accordingly to resting echo parameters. The results observed with Doppler evaluation during and after exercise test clearly can influence the classification of the pathology—obstructive versus nonobstructive—and the therapeutic intervention. It is a fact that the exercise test better reproduces 24-hours performance and common activities developed by patients in their lives, and so it should complement resting evaluation. Besides, it helps us to better understand cardiovascular behavior and physiology triggered by exercise in the presence of HCM.
